# Liver Transplantation in a Child With Crigler-Najjar Syndrome Type I: A Case Report With Review of the Literature

**DOI:** 10.7759/cureus.42064

**Published:** 2023-07-18

**Authors:** Faisal A Alharbi, Nawaf R Al-Shammari, Khaled M Aloqeely

**Affiliations:** 1 Medicine, King Saud University, Riyadh, SAU; 2 Pediatric Gastroenterology, King Khalid University Hospital, Riyadh, SAU

**Keywords:** liver transplant, mutation, ugt1a1, unconjugated bilirubin, autosomal recessive, crigler-najjar syndrome

## Abstract

Crigler-Najjar syndrome (CNS) type I is a rare genetic disease caused by mutations in the UGT1A1 gene, resulting in a lack of Uridine 5'-diphospho-glucuronosyltransferase (UDPGT) enzyme. This enzyme is responsible for the glucuronidation and elimination of unconjugated bilirubin from the body. Here we report a two-month-old Saudi girl who presented with persistent unconjugated hyperbilirubinemia, reaching levels as high as 30 mg/dL despite ineffective phototherapy. The diagnosis was confirmed through sequencing, and the patient underwent a successful liver transplant at the age of two months. At the one-year follow-up, the patient is doing well. This case highlights the significance of early detection and appropriate management of CNS, emphasizing the need for prompt intervention to improve patient outcomes and prevent complications. While phototherapy offers some benefits, liver transplantation remains the only definitive treatment for this condition.

## Introduction

Both Crigler-Najjar syndrome (CNS) type I and CNS type II are hereditary conditions characterized by elevated levels of unconjugated bilirubin due to mutations in the UGT1A1 gene located on chromosome 2q37 [1]. CNS type 1 is inherited as an autosomal recessive disorder affecting bilirubin conjugation [1-3]. It typically manifests in the neonatal period and is characterized by a significant reduction or complete absence of UGT1A1 enzyme activity [1-3]. In contrast, CNS type 2 is caused by mutations that result in some residual UGT1A1 enzyme activity [1-5].

Crigler-Najjar type I is associated with severe jaundice, neurological impairment, and long-term neurological consequences [4-7]. On the other hand, individuals with CNS type 2 can survive into adulthood without neurological damage, and the severity of hyperbilirubinemia is less pronounced. Previous studies have not extensively linked CNS with other conditions such as cephalhematoma, intestinal perforation, and peritonitis [6,8].

## Case presentation

A two-month-old Saudi female infant with a clinical diagnosis of CNS type 1 was referred for consideration for a liver transplant. She was the first child of second-degree consanguineous parents, and there was a family history of sudden death at two months old due to severe jaundice of unknown cause. The pregnancy was uncomplicated, and she was delivered at full term via spontaneous vaginal delivery, weighing 3.5 kg. Jaundice was noted at birth, and phototherapy was initiated. Despite two weeks of treatment, unconjugated hyperbilirubinemia persisted, with conjugated bilirubin levels below 0.7 mg/dL and unconjugated bilirubin levels ranging from 22 to 25 mg/dL. No hemolysis or hepatic dysfunction was observed. The infant was receiving both breast milk and formula and was meeting developmental milestones appropriately for her age. Phototherapy and a trial of phenobarbital were initiated at a local hospital. However, due to persistent unconjugated hyperbilirubinemia reaching 30 mg/dL, a diagnosis of type 1 CNS was highly suspicious and exchange transfusions were performed to maintain serum bilirubin levels below the threshold for bilirubin encephalopathy (approximately 150 μmol/L or 8.7 mg/dL), along with 16-20 hours of daily phototherapy. At the time of referral, the total bilirubin level was maintained at 8 mg/dL (140 μmol/L). The infant was then started on standard 12-16 hours of phototherapy at our hospital.

At two months old, the infant presented with visible jaundice, but her systemic examination was unremarkable. She measured 55 cm in height and weighed 4.6 kg, both at the 50th percentile. Her developmental milestones were appropriate for her age.

Other than the previously mentioned bilirubin levels, the results of her complete blood count, coagulation profile, alanine transaminase (ALT), aspartate transaminase (AST), serum albumin, and liver imaging (including liver ultrasound, Doppler, and MRI brain conducted according to the protocol) were unremarkable.

Standard methods were used to collect the index case's genomic DNA. Sanger sequencing was performed for the patient on all purified PCR products. Paired-end sequencing was performed and subjected to sequence analysis. The results of sequencing presented in Table 1.

**Table 1:** Sequence variants

GENE	VARIANT COORDINATES	AMINO ACID CHANGE	SNP IDENTIFIER	ZYGOSITY	IN SILICO PARAMETERS	ALLELE FREQUENCIES	TYPE AND CLASSIFICATION
UGT1A1	NM_000463.2:c.1070A>G	p.(Gln357Arg)	Rs72551351	Homozygous	PolyPhen: Possibly damaging Align-GVGD:C15 SIFT:- Mutation Taster: Disease causing Conservation_nl:high Conservation_aa: Weak	gnomAD:0.0000040 ESP:- 1000 G: 0.0000041 CentoMD: 0.00028	Missense Likely Pathogenic (class 2)

**Figure 1:**
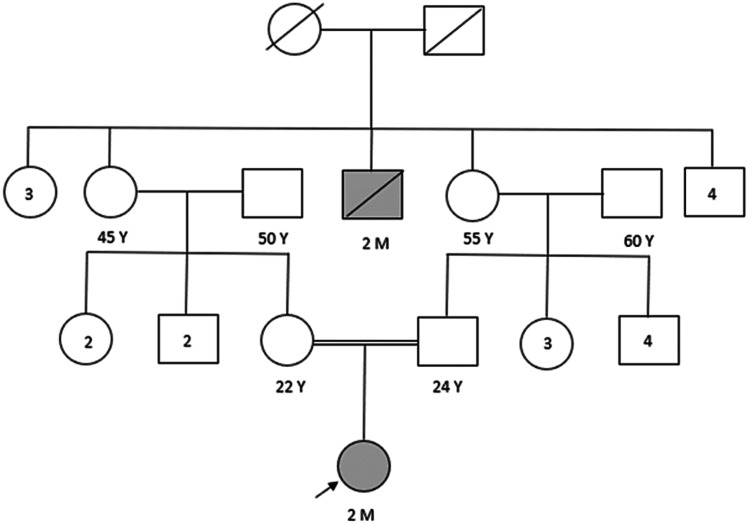
A detailed pedigree of the family

A family history was obtained, and a detailed pedigree is shown in Figure 1. Our patient was born to second-degree consanguineous parents. Among the three generations, one child died of severe jaundice at the age of two months from the mother's side.

Following that, despite the phototherapy and a trial of phenobarbital to manage the unconjugated hyperbilirubinemia. The bilirubin levels remained persistently high, reaching up to 30 mg/dL. Due to the inability to effectively manage the bilirubin levels with conservative measures the infant's father donated a left lateral liver segment for transplantation. The surgery was successful without complications, and the infant's bilirubin levels normalized post-transplantation, with unconjugated bilirubin levels at 0.64 mg/dL and conjugated bilirubin at 0.26 mg/dL. A multidisciplinary team provided post-surgical monitoring. After four weeks of hospitalization, the infant was discharged with regular follow-up visits, including blood tests for CBC, hepatic profile, renal profile, bone profile, and infliximab levels. The frequency of follow-up visits gradually decreased from weekly to every three months and the infant's condition remains stable at the one-year follow-up.

## Discussion

CNS is a rare inherited metabolic disorder characterized by a deficiency or reduced levels of the UDP-glucuronosyltransferase enzyme, which is caused by mutations in the UGT1A1 gene [1-5]. There are two types of CNS: type I, the more severe form, and type II, the milder form [1-5]. Type I CNS is characterized by a complete absence of the UGT1A1 enzyme and typically presents in infants at birth. Unfortunately, only a small percentage of infants with type I CNS survive past infancy due to the development of bilirubin encephalopathy [4,5]. In contrast, type II CNS is associated with a partial deficiency of the UGT1A1 enzyme and has a better prognosis, with a longer life expectancy [9-11]. The serum bilirubin levels in type II CNS patients range from 10 to 20 mg/dL (175 to 350 µmol/L), and the risk of kernicterus is rare. However, certain factors such as infections, fasting, or certain medications can trigger neurological complications in individuals with type II CNS [12-15]. Even though there are reported variations in the inheritance form of CNS, CNS is mostly autosomal recessive [15-22]. It is crucial to differentiate between CNS types I and II, as the treatment approaches differ. Phenobarbital, a medication that reduces blood bilirubin levels, can be effective for type II CNS but is ineffective for type I CNS [8,11]. Therefore, a lack of response to phenobarbital is a significant diagnostic indicator. Studies conducted worldwide indicate that both types of CNS have an estimated prevalence of one per million live births and affect individuals of all races and genders similarly [12-16].

Furthermore, we conducted a thorough review and collated all CNS types I and II cases, considering their disease onset, types of disease, laboratory result/treatment, clinical features, age, and gender. Moreover, we conducted a thorough literature search on Pubmed using the terms “Crigler Najjar syndrome,” “Kernicterus,” and “outcomes.”

In our case, a two-month-old Saudi female infant with persistent unconjugated hyperbilirubinemia. Despite attempts with phototherapy and a trial of phenobarbital, her bilirubin levels reached up to 30 mg/dL. She underwent exchange transfusions to keep her serum bilirubin levels below the threshold for kernicterus. Consequently, the diagnosis was confirmed through sequencing, which revealed mutations in the UGT1A1 gene associated with CNS type 1. She was urgently referred for a liver transplant. Following the transplant, the infant received immunosuppression treatment and regular follow-up, which contributed to her growth and reaching developmental milestones that are typical for her age.

## Conclusions

This case report focuses on the importance of early disease detection, as early, appropriate management is required to enhance patient outcomes and avoid complications. Phototherapy is very beneficial for these patients, but the only definitive treatment is liver transplantation.
